# Prevalence of Preeclampsia in Brazil: An Integrative Review

**DOI:** 10.1055/s-0042-1742680

**Published:** 2022-02-09

**Authors:** José Paulo de Siqueira Guida, Beatriz Gadioli de Andrade, Luis Gabriel Ferreira Pissinatti, Bruna Fagundes Rodrigues, Caio Augusto Hartman, Maria Laura Costa

**Affiliations:** 1 Departament of Tocoynecology, Faculdade de Ciências Médicas, Universidade Estadual de Campinas, Campinas, SP, Brazil; 2 Faculdade de Medicina e Odontologia e Centro de Pesquisas Odontológicas São Leopoldo Mandic, Campinas, SP, Brazil

**Keywords:** preeclampsia, epidemiology, Brazil, pré-eclâmpsia, epidemiologia, Brasil

## Abstract

**Objective** To review literature and estimate the occurrence of preeclampsia and its complications in Brazil.

**Methods** We performed an integrative review of the literature, and included observational studies published until August 2021 on the SciELO and PubMed databases that evaluated preeclampsia among pregnant women in Brazil. Other variables of interests were maternal death, neonatal death, hemolysis, elevated liver enzymes, and low platelet count (HELLP) syndrome, and eclampsia. Three independent reviewers evaluated all retrieved studies and selected those that met inclusion criteria. A metanalysis of the prevalence of preeclampsia and eclampsia was also performed, to estimate a pooled frequency of those conditions among the studies included.

**Results** We retrieved 304 studies after the initial search; of those, 10 were included in the final analysis, with a total of 52,986 women considered. The pooled prevalence of preeclampsia was of 6.7%, with a total of 2,988 cases reported. The frequency of eclampsia ranged from 1.7% to 6.2%, while the occurrence of HELLP syndrome was underreported. Prematurity associated to hypertensive disorders ranged from 0.5% to 1.72%.

**Conclusion** The frequency of preeclampsia was similar to that reported in other international studies, and it is increasing in Brazil, probably due to the adoption of new diagnostic criteria. The development of a national surveillance network would be essential to understand the problem of hypertensive disorders of pregnancy in Brazil.

## Resumo

**Objetivo** Revisar a literatura e estimar a ocorrência de pré-eclâmpsia e suas complicações no Brasil.

**Métodos** Foi realizada uma revisão integrativa da literatura, com a inclusão de estudos observacionais publicados até agosto de 2021, nas bases de dados PubMed e SciELO, que avaliavam pré-eclâmpsia em mulheres brasileiras. Outras variáveis de interesse foram morte materna, morte neonatal, síndrome de hemólise, enzimas hepáticas elevadas e plaquetopenia ( *hemolysis, elevated liver enzymes, and low platelet count* , HELLP, em inglês) e eclâmpsia. Três revisores independentes avaliaram os estudos identificados e selecionaram aqueles que preenchiam os critérios de inclusão. Foi realizada uma meta-análise da prevalência de pré-eclâmpsia e eclâmpsia, para estimar sua frequência acumulada com relação aos estudos incluídos.

**Resultados** Foram identificados 304 estudos, 10 dos quais foram incluídos na análise final, num total de 52.986 mulheres. A frequência acumulada de pré-eclâmpsia foi de 6,7%, com um total de 2.988 casos. A frequência de eclâmpsia variou de 1,7% a 6,2%, ao passo que a ocorrência de síndrome de HELLP foi pouco relatada. A prematuridade associada a hipertensão foi de 0,5% a 1,7%.

**Conclusão** A frequência de pré-eclâmpsia foi similar à de estudos internacionais; no entanto, ao longo dos últimos anos, ela vem aumentando no Brasil, possivelmente como reflexo da adoção de novos critérios diagnósticos. A criação de uma rede nacional de vigilância seria fundamental para entender o problema da hipertensão na gestação no país.

## Introduction

Preeclampsia is one of the main causes of maternal mortality and morbidity in Brazil, and adequate care may significantly reduce the mortality index related to the complications of this disease. 1 2 In Latin America, preeclampsia affects 2% to 8% of all pregnancies, and is responsible for one quarter of all maternal deaths in the region. 3 Preeclampsia and other hypertensive syndromes also determine most of medically-indicated preterm deliveries, and prematurity-related complications are the main cause of death until the fifth year of life in Brazil. 4

Preeclampsia also affects other dimensions of the daily life of women, as well as functioning during pregnancy and after childbirth. While some women die from hypertensive disorders during pregnancy, many survive, but suffer the long-term effects of the disease. 5 The overall reproductive cycle of the survivors is affected by negative or positive experiences during pregnancy, and this can affect their whole life. 6

The clinical presentation of preeclampsia may vary, but some women experience multiorgan damage and severe complications related to the disease. 7 Preeclampsia is diagnosed after twenty weeks of pregnancy in the presence of hypertension (blood pressure ≥ 140 × 90 mmHg) and proteinuria, or clinical or laboratorial evidence of organ dysfunction. 8

Studies have tried to assess frequency of preeclampsia and its complications in different settings and regions in Brazil, a country known for its regional disparities. To best of our knowledge, there is no review trying to combine data from different studies in the country. Therefore, we aimed to conduct an integrative review of original studies that evaluated the occurrence of preeclampsia in Brazil and the maternal and perinatal complications related with this condition, such as hemolysis, elevated liver enzymes, and low platelet count (HELLP) syndrome.

## Methods

We included studies that evaluated preeclampsia in different settings in Brazil. The occurrence of preeclampsia was considered according to the diagnostic criteria established in the original studies included.

Original cohorts and cross-sectional studies were included in the review. Studies that evaluated the occurrence of preeclampsia among communities or in hospital settings were included. We included studies available in PubMed and SciELO databases published until August 2021, and we excluded letters to editors, editorials, and other reviews.

For the search, we used the following terms: *preeclampsia* ; *eclampsia* ; *HELLP syndrome* ; *hypertension in pregnancy* ; *hypertensive disease in pregnancy* ; *hypertensive disorders in pregnancy* ; *observational studies* ; *cohort* ; *cross-sectionional* ; *Brazil* ; and *Latin America* . The syntax of the database search is provided below.

Syntax: ((((((((((preeclampsia) OR eclampsia) OR hellp syndrome) OR hypertension in pregnancy) OR hypertensive disease in pregnancy))) OR hypertensive disorders in pregnancy)) AND (((observational study) OR cohort) OR cross section)) AND ((brazil) OR latin america).

We also checked the reference list of each article included to obtain other articles that otherwise would not have been identified through the syntax methods.

All articles identified were included in an Excel (Microsoft Corp., Redmond, WA, United States) spreadsheet, with the identification of the authors, the year of publication, and the links to the pages. We also included the type of study, the number of participants, the setting, and the frequency of the following outcomes: preeclampsia, maternal death, neonatal death, HELLP syndrome, and eclampsia. Other relevant outcomes available were also recorded. If any data in the articles was unclear, the authors of the original studies were contacted for clarification.

We also performed a meta-analysis of the prevalence of preeclampsia and eclampsia reported in each study using a random-effect estimate. We obtained the I ^2^ rate to assess the statistical heterogeneity of the included results; an I ^2^ < 50% is considered adequate, while I ^2^ > 75% denotes substantial heterogeneity. We applied the MetaXL tool (EpiGear International, Queensland, Australia) for Excel to obtain the estimate. 9

Three independent reviewers performed the initial screening and selection of studies, considering titles and abstracts. Then, the selected studies were evaluated according to the eligibility criteria. If there were disagreements among the three reviewers, a supervisor of the study solved it and decided if the study met eligibility criteria.

To evaluate the quality of the studies included, we used the Quality Assessment Tool for Observational Cohort and Cross-Section Studies of the National Hearth, Lung and Blood Institute of the United States Department of Health and Human Services. This tool is composed by 14 questions that evaluate the methodological issues of each study included, and they also evaluate how results are presented. Each item must be answered with “Yes,” “No,” or “Not available.” The results of the evaluation must be displayed in a table to enable readers to perform an easy evaluation of the overall quality of the studies included.

## Results

**Fig. 1:**
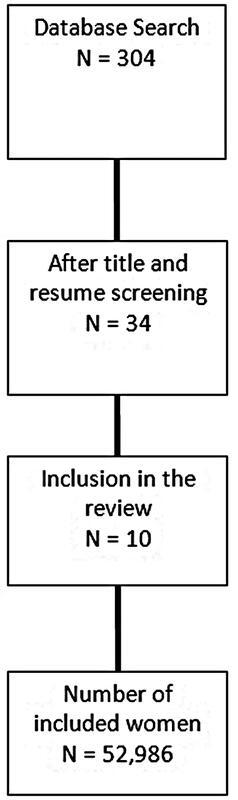
Flowchart of the inclusion of studies in the present integrative review.

A total of 304 studies were retrieved in the initial search. Out of the 287 studies retrieved from PubMed, 31 were selected after screening the titles and abstracts, and out of the 17 studies retrieved from SciELO, 1 was included. Those articles were read in full considering the inclusion and exclusion criteria were considered. Then, ten articles 10 11 12 13 14 15 16 17 18 19 were included in the present review. Figure 1 shows the inclusion flowchart.

Table 1 summarizes the results of each study included. In total, 4 presented data obtained before 2010, and 6, after 2010. A total of 52,986 women were included in the present review. None of the articles described the occurrence of maternal death, and none of the studies described all the variables of interest of the present review.

**Table 1:** Summary of the results of the studies included in the integrative review

Author (year)	Setting	N	Preeclampsia	Eclampsia	HELLP syndrome	Perinatal death	Prematurity
Gaio et al. (2001) 11	Manaus, Fortaleza, Salvador, Rio de Janeiro, São Paulo, and Porto Alegre	4,892	113	7	Not reported	Not reported	Not reported
Gonçalves et al. (2005) 12	São Paulo	604	22	1	Not reported	7	3
Dantas et al. (2013) 13	Natal	212	30	Not reported	1	Not reported	Not reported
Wendland et al. (2008) 14	Porto Alegre, São Paulo, Rio de Janeiro, Salvador, Fortaleza, and Manaus	4,766	148	Not reported	Not reported	Not reported	Not reported
Reichelt et al. (2017) 15	Porto Alegre	591	65	Not reported	Not reported	Not reported	Not reported
Mayrink et al. (2019) 10	Campinas, Botucatu, Porto Alegre, and Fortaleza	1,165	87	Not reported	Not reported	Not reported	20
Ramos Filho e Antunes (2020) 19	Belo Horizonte	36,724	2,171	36	115	Not reported	Not reported
Sanchez et al. (2021) 16	Campinas	3,102	258	Not reported	Not reported	Not reported	Not reported
Trindade et al. (2021) 17	Vitória	196	12	Not reported	Not reported	Not reported	Not reported
De Lima et al. (2021) 18	Ribeirão Preto	734	82	Not reported	Not reported	Not reported	Not reported
Total	***	52,986	2,988	***	***	***	***

Abbreviation: HELLP, hemolysis, elevated liver enzymes, and low platelet count.

Overall, 2,988 cases of preeclampsia were reported; the prevalence ranged from 2.31% 11 to 14.15%. 13 Seven studies 10 13 14 15 16 17 18 did not report the occurrence of eclampsia. In the other 3 studies that reported eclampsia, it ranged from 1.66% 19 to 6.19%. 11 Only one case of HELLP syndrome was reported in one study, 13 which also reported a frequency of 30 cases of preeclampsia.

One study 12 reported 7 perinatal deaths among a group of 604 women followed up due to hypertensive disorders. Two studies 10 12 reported a prematurity ratio ranging from 0.5% to 1.72%.

To estimate the overall prevalence of preeclampsia, all studies included were considered for the prevalence meta-analysis. The pooled frequency of preeclampsia was of 6.7% (confidence interval [CI]: 4.9–8.6%; I ^2^ = 97.3%). We also found that the prevalence of preeclampsia increased when comparing two different periods. The pooled preeclampsia prevalence increased from 4.4% (CI: 2.6–6.8%; I ^2^ = 93.8%) in studies published until 2010 to 8.2% (CI: 6.4–10.2%, I ^2^ = 92.9%) in studies published after 2010.

**Fig. 2:**
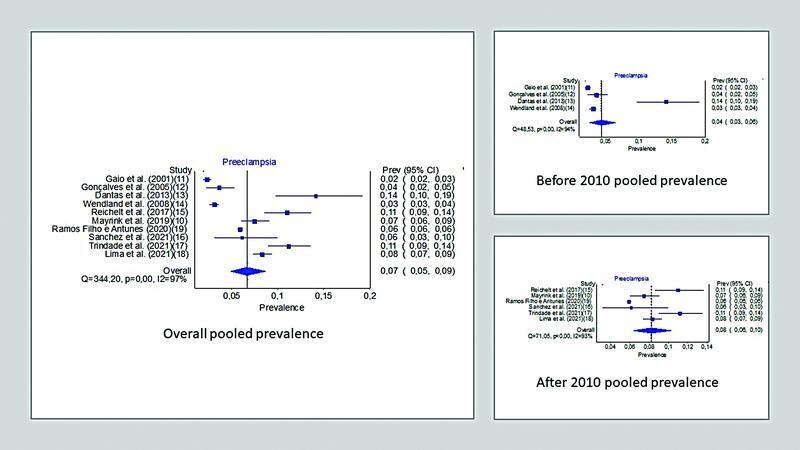
Forest plots of the pooled prevalence of preeclampsia (total study period, before 2010, and after 2010).

Figure 2 presents the three forest plots with pooled frequencies of preeclampsia.

We also estimated the pooled prevalence of eclampsia regarding the three studies 11 12 19 that reported cases of it. Our metanalysis showed a prevalence of 3.3% (CI: 0.3–8.5%; I ^2^ = 76.3%). All of the ten studies included had good methodological quality, and Table 2 presents a quality assessment of them.

**Table 2:** Quality assessment tool for observational-cohort and cross-sectional studies

Study/Question	1	2	3	4	5	6	7	8	9	10	11	12	13	14
Gaio et al. (2001) 11	Yes	Yes	Yes	Yes	No	Yes	Yes	No	Yes	No	Yes	No	Yes	Yes
Gonçalves et al. (2005) 12	Yes	Yes	No	Yes	No	Yes	Yes	No	Yes	No	Yes	No	Yes	Yes
Dantas et al. (2013) 13	Yes	Yes	Yes	Yes	Yes	Yes	Yes	No	Yes	Yes	Yes	No	Yes	Yes
Wendland et al. (2008) 14	Yes	Yes	Yes	Yes	Yes	Yes	Yes	No	Yes	No	No	No	Yes	Yes
Reichelt et al. (2017) 15	Yes	Yes	Yes	Yes	Yes	Yes	Yes	No	Yes	No	Yes	No	Yes	Yes
Mayrink et al. (2019) 10	Yes	Yes	Yes	Yes	Yes	Yes	Yes	No	Yes	Yes	Yes	No	Yes	Yes
Ramos Filho e Antunes (2020) 19	Yes	Yes	Yes	Yes	Yes	Yes	Yes	No	Yes	No	Yes	No	Yes	Yes
Sanchez et al. (2021) 16	Yes	Yes	Yes	Yes	Yes	Yes	Yes	No	Yes	No	Yes	No	Yes	Yes
Trindade et al. (2021) 17	Yes	Yes	Yes	Yes	Yes	Yes	Yes	No	Yes	No	Yes	No	Yes	Yes
De Lima et al. (2021) 18	Yes	Yes	Yes	Yes	Yes	Yes	Yes	No	Yes	No	Yes	No	Yes	Yes

## Discussion

Our results show that there are few population-based studies published in international databases specifically on preeclampsia in Brazil. Probably due to the costs associated to perform a population-based investigation, most studies that evaluated preeclampsia in Brazil were cross-sectional, a study design that does not enable the estimation of the incidence.

The overall frequency of preeclampsia in Brazil was similar to that reported elsewhere; 20 however, some Brazilian studies 21 presented frequencies much higher than those reported in international studies.

The frequency of the other outcomes studied was low; however, our results cannot be generalized, because we cannot state that the absence of data reflects the absence of occurrence of the outcomes. There was no standardization in the presentation of outcomes in the studies included in the present review. An effort to disseminate a consensual set of outcomes for hypertension-related studies would facilitate the collection and metanalysis of results from different studies and datasets. 22

Our results also show an increase in the frequency of preeclampsia from 2010, when new diagnostic criteria for preeclampsia were established and widely accepted, 7 until today. Among the 6 studies included that were conducted after 2010, 5 followed the new guidelines for preeclampsia, suggesting that the disease was probably underdiagnosed before 2010, or that the new diagnostic criteria can identify women with preeclampsia more adequately.

Even after the expansion of the diagnostic criteria, we did not observ an increase in the occurrence of eclampsia or maternal death. Many studies suggest that adequate blood pressure control and maternal and perinatal surveillance are related to better maternal and perinatal results. Broader diagnostic criteria may impact surveillance. 23

A limitation of the present review is that few studies were included; this, however, reflected the difficulties in performing populational studies in our country. The deveopment of national databases to follow up women during pregnancy and the postpartum period, in a prospective and collaborative way, will help us to better understand the experiences of women throughout their reproductive lives.

## Conclusion

In the Brazilian studies included in the present review, the frequencies of preeclampsia and prematurity, but not of perinatal death, were similar to those reported in international studiess. The standardization of preeclampsia-related results is key to the adequate collection of results. The frequency of preeclampsia is probably increasing in Brazil due to new diagnostic criteria and higher surveillance. The development of national networks of research on preeclampsia is paramount to understand the impact of the disease in Brazil.
